# Quality of Life and Psychological Factors in Patients with Metastatic Prostate Cancer Receiving Androgen Receptor–Targeted Therapies: A Prospective Cross-Sectional Real-World Study

**DOI:** 10.3390/medicina62061175

**Published:** 2026-06-17

**Authors:** Selahattin Çelik, Salih Karatlı, Mehmetcan Atak, Hatice Ayyıldız Sevim, Gökşen İnanç İmamoğlu, Samed Rahatlı

**Affiliations:** Department of Medical Oncology, Ankara Etlik City Training and Research Hospital, 06010 Ankara, Turkey; karatlisalih@hotmail.com (S.K.);

**Keywords:** metastatic prostate cancer, quality of life, androgen receptor-targeted therapy, anxiety, depression, patient-reported outcomes

## Abstract

*Background and Objectives*: Quality of life (QoL) has become an essential outcome in patients with metastatic prostate cancer, particularly in the era of androgen receptor (AR)-targeted therapies. Although these agents improve survival, their differential impact on QoL and the role of psychological factors remain incompletely understood. This study aimed to evaluate QoL, functional outcomes, and psychological status, and to identify factors associated with poor QoL in a real-world cohort. *Materials and Methods*: This prospective cross-sectional, single-center observational study included 130 patients with metastatic prostate cancer receiving AR-targeted therapies (abiraterone, enzalutamide, or apalutamide/darolutamide). QoL was assessed using the EORTC QLQ-C30 questionnaire, and psychological status was evaluated using the Hospital Anxiety and Depression Scale (HADS). Patients were stratified according to treatment groups, and comparisons were performed using appropriate statistical tests. Logistic regression analyses were conducted to determine factors independently associated with poor QoL. *Results*: Exploratory differences in global QoL were observed among treatment groups (*p* = 0.007), with lower global QoL scores in the abiraterone group and numerically higher emotional and cognitive functioning scores in the enzalutamide group. Symptom analysis demonstrated higher nausea/vomiting scores in the abiraterone group (*p* = 0.022), whereas other symptom domains were comparable across treatment groups. In multivariable analysis, anxiety (odds ratio [OR]: 6.62) and depression (OR: 3.40) were independently associated with poor QoL, while treatment type was not independently associated with poor QoL after multivariable adjustment. *Conclusions*: Although unadjusted QoL scores differed across AR-targeted therapy groups, psychological factors—particularly anxiety and depression—were significantly associated with poorer QoL in patients with metastatic prostate cancer. These findings highlight the importance of integrating routine psychosocial assessment and supportive care strategies into clinical practice to optimize patient-centered outcomes. However, given the cross-sectional and exploratory nature of the study, the findings should be interpreted cautiously.

## 1. Introduction

Prostate cancer is one of the most commonly diagnosed malignancies and remains a leading cause of cancer-related mortality among men worldwide, representing a major global health burden despite advances in screening and treatment strategies [1]. While many patients are diagnosed with localized disease, a substantial proportion eventually develop metastatic prostate cancer, which is associated with significantly worse prognosis and increased symptom burden [2].

Over the past decade, the therapeutic landscape of metastatic prostate cancer has evolved substantially with the introduction of androgen receptor (AR)-targeted therapies, including abiraterone acetate, enzalutamide, apalutamide, and darolutamide [3]. These agents have demonstrated significant improvements in radiographic progression-free and overall survival across both metastatic castration-sensitive and castration-resistant disease settings, establishing them as a cornerstone of systemic treatment in contemporary clinical practice [4,5,6,7].

As survival outcomes improve, attention has increasingly shifted toward patient-reported outcomes, particularly quality of life (QoL), which reflects the broader impact of disease and treatment on physical, emotional, and social functioning [8,9]. Previous randomized clinical trials and real-world studies have suggested that AR-targeted therapies differ in their toxicity profiles, including fatigue, cognitive impairment, and emotional disturbances, which may translate into clinically meaningful differences in QoL outcomes among treatment groups [10].

Importantly, psychological conditions, particularly anxiety and depression, are highly prevalent among patients with metastatic cancer and have been increasingly recognized as important factors associated with quality of life and overall clinical outcomes [11]. Several studies have demonstrated that psychological distress may be associated with poorer QoL, reduced treatment adherence, and even inferior survival outcomes in oncology populations [9,12]. Despite the growing recognition of the importance of QoL and psychological well-being, most available data are derived from randomized clinical trials, which may not fully reflect real-world clinical practice due to strict inclusion criteria and selected patient populations [13,14]. Moreover, direct comparisons of QoL outcomes among different AR-targeted therapies in real-world settings remain limited, and the relative contribution of psychological factors to QoL in this population remains incompletely characterized. Other psychosocial factors, including social support, coping strategies, caregiver burden, and socioeconomic conditions, may also substantially influence QoL outcomes in patients with metastatic cancer.

Therefore, the present study aimed to comprehensively evaluate quality of life, functional outcomes, and psychological status in patients with metastatic prostate cancer receiving AR-targeted therapies, including abiraterone, enzalutamide, and apalutamide/darolutamide, in a real-world setting. In addition, we sought to evaluate factors associated with poor QoL, with a particular focus on the impact of anxiety and depression in this patient population.

## 2. Materials and Methods

### 2.1. Study Design and Participants

This prospective cross-sectional, single-center observational study was conducted among patients with metastatic prostate cancer who were followed at the medical oncology outpatient clinic. A total of 130 patients receiving androgen receptor (AR)-targeted therapies (abiraterone, enzalutamide, or apalutamide/darolutamide) during the study period were included. Among the 13 patients included in the apalutamide/darolutamide subgroup, 10 patients received apalutamide and 3 received darolutamide. Due to the limited sample size, analyses involving this subgroup were considered exploratory and should be interpreted with caution. All included patients had been receiving AR-targeted therapy for at least 6 months prior to study enrollment to ensure sufficient treatment exposure for reliable assessment of quality of life.

Eligible patients were required to be 18 years of age or older, have a confirmed diagnosis of metastatic prostate cancer, be receiving one of the specified AR-targeted therapies, and be capable of completing the study questionnaires. Patients with incomplete clinical data, inability to complete the questionnaires, or severe cognitive impairment were excluded.

**Figure 1:**
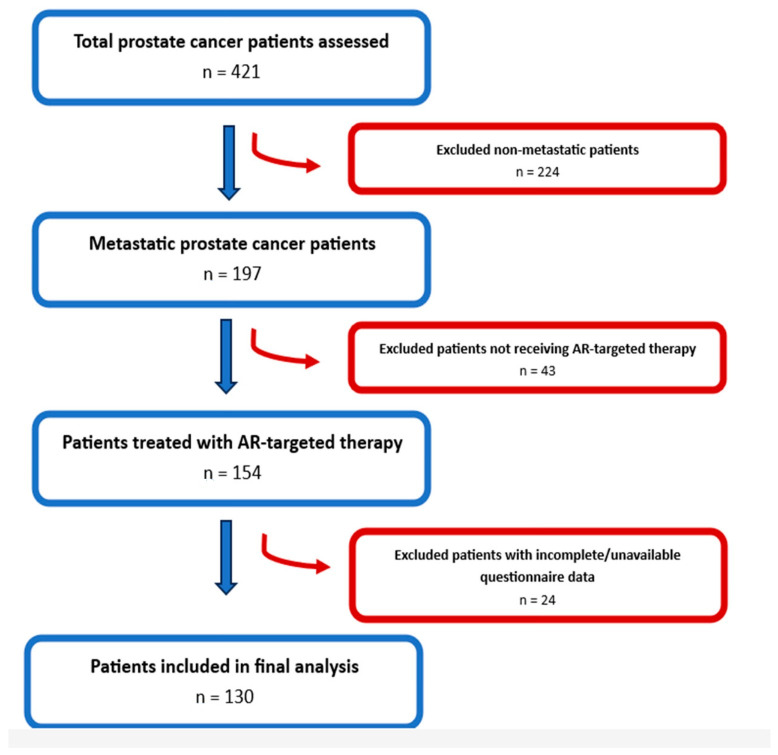
Flow diagram of patient selection and study inclusion.

During the study period, a total of 421 patients with prostate cancer were assessed for eligibility. Of these, 224 patients without metastatic disease were excluded, leaving 197 patients with metastatic prostate cancer. Among these patients, 43 were not receiving AR-targeted therapy and were therefore excluded. Of the remaining 154 patients treated with AR-targeted therapy, 24 were excluded because of incomplete or unavailable questionnaire data. Ultimately, 130 patients were included in the final analysis (Figure 1).

### 2.2. Ethical Considerations

This study was conducted in accordance with the principles of the Declaration of Helsinki. Written informed consent was obtained from all participants prior to enrollment. Ethical approval was obtained from the institutional ethics committee of Ankara Etlik City Hospital (AEŞH-BADEK2-2025-402).

### 2.3. Data Collection and Measures

Sociodemographic and clinical data were collected using a structured questionnaire and patient medical records. Collected variables included age, marital status, education level, income status, and clinical characteristics.

Quality of life (QoL) was assessed using the European Organisation for Research and Treatment of Cancer Quality of Life Questionnaire (EORTC QLQ-C30). This validated instrument evaluates global QoL, functional domains (physical, role, emotional, cognitive, and social functioning), and symptom scales. Scores range from 0 to 100; higher scores indicate better global health status and functioning, whereas higher symptom scores indicate greater symptom burden. A 10-point difference in EORTC QLQ-C30 scores was considered clinically meaningful, in accordance with commonly used interpretation criteria for this instrument.

Psychological status was evaluated using the Hospital Anxiety and Depression Scale (HADS), which consists of two subscales: anxiety (HADS-A) and depression (HADS-D). A cut-off value of ≥8 for each HADS subscale was used to define clinically significant anxiety and depression, as this threshold is commonly applied for screening purposes in oncology populations.

Performance status was assessed using the Eastern Cooperative Oncology Group (ECOG) performance status scale.

### 2.4. Study Variables

Poor QoL was defined as a global QoL score <60, corresponding to at least a 10-point decrease below the study population mean global QoL score of approximately 70. This threshold was used for exploratory categorization purposes and was based on the commonly accepted 10-point clinically meaningful difference in EORTC QLQ-C30 scores. However, the applicability of this threshold may vary across different patient populations and clinical settings.

Independent variables included age, marital status, education level, income status, ECOG performance status, anxiety, and depression.

### 2.5. Statistical Analysis

Statistical analyses were performed using IBM SPSS Statistics version 25.0 (IBM Corp., Armonk, NY, USA).

Continuous variables were presented as mean ± standard deviation (SD) or median (minimum–maximum), as appropriate, while categorical variables were expressed as frequencies and percentages.

Normality of continuous variables was assessed using the Shapiro–Wilk test.

Continuous variables were compared using one-way analysis of variance (ANOVA) or the Kruskal–Wallis test according to data distribution. Categorical variables were analyzed using the chi-square test or Fisher’s exact test, as appropriate.

To identify factors associated with poor QoL, logistic regression analysis was performed. Univariable analyses were initially conducted, and variables with a *p*-value < 0.10 were included in the multivariable model. Results were reported as odds ratios (ORs) with 95% confidence intervals (CIs). Given the relatively limited sample size, the multivariable analysis was considered exploratory.

A two-sided *p*-value < 0.05 was considered statistically significant.

Because of the exploratory nature of the study, formal correction for multiple comparisons was not applied, and findings should therefore be interpreted cautiously.

## 3. Results

### 3.1. Patient Characteristics

A total of 130 patients with metastatic prostate cancer were included in the study. Of these, 59 (45.4%) received abiraterone, 58 (44.6%) enzalutamide, and 13 (10.0%) apalutamide/darolutamide.

The median age of the study population was 71 years (range: 30–89), and a statistically significant difference in age was observed among treatment groups (*p* = 0.023), with patients in the enzalutamide group being older. No significant differences were found between groups in terms of marital status, education level, or income status (*p* = 0.633, *p* = 0.862, and *p* = 0.444, respectively). ECOG performance status differed significantly across treatment groups (*p* = 0.040); however, this finding should be interpreted cautiously because of the limited number of patients with ECOG ≥ 2. Detailed demographic and clinical characteristics are presented in Table 1.

### 3.2. Quality of Life and Functional Outcomes

Exploratory differences in global QoL scores were observed among treatment groups (*p* = 0.007). Patients receiving abiraterone had lower global QoL scores (65.1 ± 19.4) compared to those receiving enzalutamide (74.1 ± 17.4) and apalutamide/darolutamide (75.0 ± 17.4). The observed differences exceeded the commonly accepted 10-point threshold considered clinically meaningful for EORTC QLQ-C30 global QoL scores.

Among functional domains, emotional functioning (*p* = 0.007) and cognitive functioning (*p* = 0.037) showed exploratory between-group differences, with numerically higher scores observed in the enzalutamide group; however, these unadjusted findings should be interpreted cautiously because baseline age and ECOG performance status differed among treatment groups. In addition, the magnitude and clinical relevance of these differences should be interpreted cautiously. No significant differences were observed in physical, role, or social functioning (all *p* > 0.05). Detailed QoL and functional outcomes are summarized in Table 2.

### 3.3. Symptom Burden

Fatigue was the most prominent symptom across all treatment groups; however, the difference was not statistically significant (*p* = 0.089). Nausea and vomiting scores differed significantly among groups (*p* = 0.022), with higher values observed in the abiraterone group.

No statistically significant differences were found for other symptoms, including pain, dyspnea, insomnia, appetite loss, constipation, diarrhea, or financial difficulties (all *p* > 0.05). Symptom scores are presented in Table 3.

### 3.4. Psychological Status

The prevalence of depression differed significantly among treatment groups (*p* = 0.045), with the highest rate observed in the abiraterone group. In contrast, the prevalence of anxiety did not differ significantly between groups (*p* = 0.077). Detailed distributions of depression and anxiety according to treatment groups are shown in Table 4.

### 3.5. Factors Associated with Poor Quality of Life

Logistic regression analysis was performed to identify factors associated with poor QoL (defined as a global QoL score <60).

In univariable analysis, age, marital status, income status, and ECOG performance status were not significantly associated with poor QoL (all *p* > 0.05). Enzalutamide was associated with a lower likelihood of poor QoL compared to abiraterone (OR: 0.39, 95% CI: 0.18–0.88, *p* = 0.023), while overall treatment type showed borderline significance (*p* = 0.057). Anxiety (OR: 11.08, 95% CI: 4.55–26.98, *p* < 0.001) and depression (OR: 7.00, 95% CI: 2.89–16.95, *p* < 0.001) were significantly associated with poor QoL in univariable analysis.

In multivariable analysis, treatment type was not independently associated with poor QoL (*p* = 0.535). These findings suggest that the apparent unadjusted differences in QoL across treatment groups may be partly explained by psychological factors and baseline patient characteristics. In contrast, anxiety and depression remained independently associated with poor QoL. Anxiety was independently associated with a higher likelihood of poor QoL (OR: 6.62, 95% CI: 2.53–17.32, *p* < 0.001), while depression was also independently associated with poor QoL (OR: 3.40, 95% CI: 1.27–9.10, *p* = 0.015). Detailed results are presented in Table 5.

## 4. Discussion

In this real-world study of patients with metastatic prostate cancer receiving AR-targeted therapies, several clinically relevant observations emerged regarding QoL and factors associated with QoL. First, exploratory differences in global QoL and specific functional domains were observed among treatment groups, with patients receiving enzalutamide showing numerically higher scores in certain QoL domains, particularly emotional and cognitive functioning. Second, psychological factors—specifically anxiety and depression assessed by HADS—were significantly associated with poor QoL. Notably, while treatment type showed an association with QoL in univariable analysis, this relationship was not maintained in multivariable models, suggesting a potentially important association between psychological distress and QoL outcomes and baseline patient characteristics over treatment-related factors.

The observed differences in QoL across AR-targeted therapies are consistent with previous reports suggesting that these agents have distinct toxicity profiles that may influence patient-reported outcomes. Fatigue, cognitive impairment, and emotional disturbances have been particularly associated with AR pathway inhibitors, and these adverse effects may translate into clinically meaningful differences in QoL [10]. However, this observation should be interpreted cautiously because the treatment groups were not randomized and differed in baseline characteristics, including age and ECOG performance status. Therefore, these findings should be considered exploratory rather than definitive evidence of treatment-related superiority [15,16].

Importantly, one of the key findings of our study is the significant association between psychological distress and poor QoL. Both anxiety and depression were independently associated with poor QoL in multivariable analysis, with anxiety showing a particularly strong association. These findings are in line with prior literature demonstrating that psychological distress is closely associated with impaired QoL in oncology populations [11]. While the association between psychological distress and QoL has been reported previously in broader oncology populations, the present study demonstrates a similar association in a real-world cohort of patients with metastatic prostate cancer receiving contemporary AR-targeted therapies. The finding that anxiety and depression remained independently associated with poor QoL after adjustment for treatment type may support the integration of routine psychosocial assessment and supportive care strategies into the management of this specific patient population.

Psychological distress may negatively affect multiple domains of QoL, including emotional, cognitive, and social functioning, and may also impair patients’ ability to cope with disease-related symptoms and treatment-related toxicities [12,17]. Future studies should additionally evaluate broader psychosocial determinants, including social support systems, coping strategies, caregiver burden, and socioeconomic influences, to provide a more comprehensive understanding of QoL determinants.

The mechanisms underlying the association between psychological distress and poor QoL are likely multifactorial. Anxiety and depression have been linked to increased symptom perception, reduced treatment adherence, and dysregulation of neuroendocrine and inflammatory pathways, all of which may contribute to worse clinical outcomes [18]. In addition, psychological distress may amplify the subjective perception of treatment-related side effects, further worsening patient-reported outcomes.

Notably, the attenuation of the treatment effect in multivariable analysis suggests that the observed differences in QoL between AR-targeted therapies may be partly driven by underlying psychological factors and baseline patient characteristics rather than the intrinsic properties of the treatments themselves. This finding suggests that patient-related factors, particularly psychological distress, may contribute to QoL outcomes beyond treatment-related effects in metastatic prostate cancer.

Our findings should be interpreted in the context of both randomized clinical trial data and real-world observational evidence to evaluate QoL outcomes. Clinical trials often include highly selected patient populations and may not fully capture the complexity of real-world clinical practice, particularly with regard to psychosocial factors [13]. This is particularly relevant for patient-reported outcomes, which are highly sensitive to psychosocial and contextual factors often underrepresented in clinical trials. Cultural and socioeconomic factors may also influence psychological distress, coping behaviors, symptom perception, and QoL assessments, potentially contributing to variability across different patient populations and healthcare settings.

From a clinical perspective, these findings emphasize the need for a multidisciplinary approach in the management of metastatic prostate cancer. Routine screening for anxiety and depression using validated tools such as HADS should be considered an integral component of patient care. Early identification and appropriate management of psychological distress may improve QoL, enhance treatment adherence, and potentially influence overall clinical outcomes [9,19].

This study has several limitations. First, the cross-sectional design of the study precludes causal inferences between psychological factors and QoL and introduces temporal ambiguity regarding the directionality of these associations. Second, the single-center nature of the study and the relatively small sample size, particularly in the apalutamide/darolutamide subgroup, may limit the generalizability of the findings. In addition, the small number of patients in the apalutamide/darolutamide subgroup reduced the statistical power for treatment-specific comparisons; therefore, findings related to this subgroup should be considered exploratory and interpreted with caution. Third, potential confounding factors, including unmeasured comorbidities, disease burden, metastatic characteristics, treatment line, prior treatments, treatment duration, and broader psychosocial variables, could not be fully accounted for. Selection bias and survivor bias may also have been present, as only patients able to complete questionnaires after at least 6 months of therapy were included. Consequently, the findings may not be fully generalizable to patients with rapidly progressive disease or severe functional impairment. In addition, cultural and socioeconomic factors that may influence psychological distress, coping behaviors, and QoL perceptions were not comprehensively evaluated. Nevertheless, the study also has important strengths, including the use of validated instruments (EORTC QLQ-C30 and HADS), a real-world patient population, and a comprehensive evaluation of both clinical and psychosocial variables. Furthermore, the prospective collection of patient-reported outcomes using validated questionnaires represents an important methodological strength of the study. In addition, multiple comparisons were performed without formal correction procedures, which may have increased the risk of type I error. In addition, the use of a dichotomized QoL outcome may have resulted in some loss of information compared with analyses using continuous QoL measures.

## 5. Conclusions

Our findings suggest that although unadjusted QoL scores may differ among AR-targeted therapy groups, psychological factors—particularly anxiety and depression—were significantly associated with poorer QoL in patients with metastatic prostate cancer. These findings highlight the importance of incorporating routine psychosocial assessment and supportive care strategies into clinical practice to improve patient-centered outcomes. However, given the cross-sectional and exploratory nature of the study, the findings should be interpreted cautiously. Further large-scale prospective longitudinal studies incorporating broader psychosocial, clinical, and socioeconomic variables are warranted to better clarify the relative contributions of treatment-related and psychological factors to QoL in this patient population. After multivariable adjustment, psychological distress appeared to have a stronger association with QoL than treatment type, highlighting the potential importance of psychosocial factors in this patient population.

## Figures and Tables

**Table 1:** Demographic and Clinical Characteristics of the Patients.

Variable	Abiraterone (n = 59)	Enzalutamide (n = 58)	Apalutamide/Darolutamide (n = 13)	*p*-Value
Age (years)	68.5 ± 10.4	72.7 ± 8.7	68.5 ± 9.7	0.023
Marital status				0.633
Married	47 (79.7)	50 (86.2)	11 (84.6)	
Single	12 (20.3)	8 (13.8)	2 (15.4)	
Education level				0.862
Primary or less	39 (66.1)	40 (69.0)	8 (61.5)	
High school/University	20 (33.9)	18 (31.0)	5 (38.5)	
Income status				0.444
Income < expenses	50 (84.7)	44 (75.9)	11 (84.6)	
Income ≥ expenses	9 (15.3)	14 (24.1)	2 (15.4)	
ECOG performance status				0.040
ECOG 0–1	58 (98.3)	50 (86.2)	11 (84.6)	
ECOG ≥ 2	1 (1.7)	8 (13.8)	2 (15.4)	

Abbreviations: ECOG, Eastern Cooperative Oncology Group.

**Table 2:** Global Quality of Life and Functional Scores.

Score	Abiraterone	Enzalutamide	Apalutamide/Darolutamide	*p*-Value
Global QoL	65.1 ± 19.4	74.1 ± 17.4	75.0 ± 17.4	0.007
Physical functioning	76.4 ± 20.9	79.8 ± 20.2	83.9 ± 19.0	0.182
Role functioning	77.3 ± 23.5	79.6 ± 19.0	63.9 ± 22.3	0.153
Emotional functioning	75.7 ± 17.6	83.2 ± 20.7	81.3 ± 24.1	0.007
Cognitive functioning	77.9 ± 19.1	85.9 ± 17.3	83.3 ± 18.8	0.037
Social functioning	71.6 ± 18.5	74.7 ± 15.4	69.4 ± 22.3	0.452

Abbreviations: QoL, quality of life; SD, standard deviation. Footnote: Data are presented as mean ± SD. Higher scores indicate better functioning and global health status.

**Table 3:** Symptom Scores.

Symptom	Abiraterone	Enzalutamide	Apalutamide/Darolutamide	*p*-Value
Fatigue	26.6 ± 23.4	20.1 ± 16.8	13.7 ± 9.2	0.089
Nausea/Vomiting	31.9 ± 27.8	25.3 ± 49.5	14.1 ± 16.5	0.022
Pain	13.5 ± 25.8	6.6 ± 12.5	3.8 ± 7.3	0.442
Dyspnea	5.7 ± 12.7	6.3 ± 17.0	5.1 ± 12.5	0.943
Insomnia	10.3 ± 19.9	17.8 ± 26.6	43.6 ± 90.7	0.118
Appetite loss	6.9 ± 15.0	6.9 ± 15.0	5.1 ± 12.5	0.911
Constipation	23.0 ± 17.9	20.1 ± 19.7	20.5 ± 16.9	0.552
Diarrhea	25.3 ± 26.0	20.1 ± 25.7	12.8 ± 16.9	0.206
Financial difficulties	25.3 ± 20.0	27.0 ± 29.6	30.8 ± 21.4	0.651

Abbreviations: SD, standard deviation. Footnote: Data are presented as mean ± SD. Higher scores indicate greater symptom burden. Because some symptom scores showed skewed distributions, between-group comparisons were performed using non-parametric tests when appropriate.

**Table 4:** Distribution of Depression and Anxiety According to Treatment Groups (HADS).

Variable	Abiraterone (n = 59)	Enzalutamide (n = 58)	Apalutamide/Darolutamide (n = 13)	*p*-Value
Depression (HADS-D ≥ 8)	37 (62.7%)	24 (41.4%)	5 (38.5%)	0.045
No depression	22 (37.3%)	34 (58.6%)	8 (61.5%)	
Anxiety (HADS-A ≥ 8)	22 (37.3%)	12 (20.7%)	2 (15.4%)	0.077
No anxiety	37 (62.7%)	46 (79.3%)	11 (84.6%)	

Abbreviations: HADS, Hospital Anxiety and Depression Scale; HADS-A, anxiety subscale; HADS-D, depression subscale. Footnote: Data are presented as n (%). A cut-off value of ≥8 was used to define clinically significant anxiety and depression.

**Table 5:** Logistic Regression Analysis for Poor Quality of Life.

Variable	Univariable OR (95% CI)	*p*-Value	Multivariable OR (95% CI)	*p*-Value
Age (continuous)	1.00 (0.96–1.04)	0.998	—	—
Marital status (single vs. married)	1.30 (0.49–3.39)	0.594	—	—
Income status (≥expenses vs. <expenses)	0.81 (0.31–2.13)	0.672	—	—
ECOG ≥ 2	0.80 (0.20–3.18)	0.751	—	—
Treatment type		0.057		0.535
Enzalutamide vs. Abiraterone	0.39 (0.18–0.88)	0.023	0.59 (0.23–1.51)	0.267
Apalutamide/Darolutamide vs. Abiraterone	0.41 (0.10–1.64)	0.206	0.71 (0.14–3.63)	0.678
Anxiety (HADS-A ≥ 8)	11.08 (4.55–26.98)	<0.001	6.62 (2.53–17.32)	<0.001
Depression (HADS-D ≥ 8)	7.00 (2.89–16.95)	<0.001	3.40 (1.27–9.10)	0.015

Abbreviations: OR, odds ratio; CI, confidence interval; ECOG, Eastern Cooperative Oncology Group. Footnote: Poor quality of life was defined as a global QoL score < 60. Variables with *p* < 0.10 in univariable analysis were considered for multivariable analysis. The association between ECOG ≥ 2 and poor QoL should be interpreted cautiously due to the small number of patients with ECOG ≥ 2. Findings related to the apalutamide/darolutamide subgroup should also be interpreted cautiously because of the limited sample size.

## Data Availability

The data presented in this study are available on reasonable request from the corresponding author.

## Abbreviations

AR	Androgen Receptor
QoL	Quality of Life
EORTC QLQ-C30	European Organisation for Research and Treatment of Cancer Quality of Life Questionnaire-Core 30
HADS	Hospital Anxiety and Depression Scale
HADS-A	Hospital Anxiety and Depression Scale—Anxiety
HADS-D	Hospital Anxiety and Depression Scale—Depression
ECOG	Eastern Cooperative Oncology Group
OR	Odds Ratio
CI	Confidence Interval
SD	Standard Deviation
PROs	Patient-Reported Outcomes
